# Neutrophils in Post-myocardial Infarction Inflammation: Damage vs. Resolution?

**DOI:** 10.3389/fcvm.2019.00025

**Published:** 2019-03-18

**Authors:** Sarah-Lena Puhl, Sabine Steffens

**Affiliations:** ^1^Institute for Cardiovascular Prevention (IPEK), Ludwig-Maximilians-University (LMU), Munich, Germany; ^2^German Centre for Cardiovascular Research (DZHK), Partner Site Munich Heart Alliance, Munich, Germany

**Keywords:** myeloperoxidase, ischemia/reperfusion, cardiac remodeling, heart failure, neutrophil subsets, circadian rhythm, sexual dimorphism

## Abstract

Inflammation not only plays a crucial role in acute ischemic cardiac injury, but also contributes to post-infarction repair and remodeling. Traditionally, neutrophils have been merely considered as detrimental in the setting of an acute myocardial infarction. However, recently published studies demonstrated that neutrophils might also play an important role in cardiac repair by regulating reparative processes. An emerging concept is that different neutrophil subsets exist, which might exhibit separate functional properties. In support of the existence of distinct neutrophil subsets in the ischemic heart, transcriptional changes in cardiac neutrophils have been reported within the first few days after myocardial infarction. In addition, there is an increasing awareness of sex-specific differences in many physiological and pathophysiological responses, including cardiovascular parameters and inflammation. Of particular interest in this context are recent experimental data dissecting sex-specific differences in neutrophil signaling after myocardial infarction. Unraveling the distinct and possibly stage-dependent properties of neutrophils in cardiac repair may provide new therapeutic strategies in order to improve the clinical outcome for myocardial infarction patients. This review will briefly discuss recent advances in our understanding of the neutrophil functional repertoire and emerging insights of sex-specific differences in post-myocardial infarction inflammatory responses.

## Introduction

Acute myocardial infarction (MI) is the leading cause of mortality and morbidity in Europe. The lack of oxygen and nutrient supply leads to a massive loss of cardiac tissue, which induces an inflammatory response that initiates cardiac repair processes (1). Attracted by cell debris and inflammatory signals released by activated neighboring cells, neutrophils massively infiltrate the infarct area in the first few hours following onset of ischemia. These cells, which are also called polymorphonuclear granulocytes, are key protagonists of the innate immune response, representing 10–25% of the circulating leukocytes in mice and 50–70% in humans (2). They generate high levels of reactive oxygen species and secrete granule components including myeloperoxidase and proteases, thereby exacerbating local vascular and tissue injury (3). Subsequently, blood monocyte-derived macrophages infiltrate the infarct area to remove cardiac tissue debris and apoptotic neutrophils, which, in turn activates reparative pathways necessary for scar formation (1). Although these innate immune cells are integral key players of cardiac healing, an unbalanced or exaggerated immune reaction after MI aggravates tissue damage that consecutively triggers maladaptive remodeling (4, 5). Macrophages in the ischemic myocardium exhibit a high plasticity and are involved in both inflammatory as well as reparative processes (6, 7). During post-MI inflammation resolution, pro-inflammatory macrophages are thought to undergo local conversion to resolution-mediating macrophages. Neutrophils seem to play crucial role in this process, as their granule content does not only contribute to ischemic damage and well-described ischemia-reperfusion injury, but also to the recruitment of monocytes and macrophage reprograming toward a resolving phenotype (8, 9). Neutrophil depletion in experimental MI results in impaired resolution of inflammation and uncoordinated fibrotic scar formation, which translates into adverse remodeling and decreased cardiac function (9). In support of this beneficial role for neutrophils during tissue repair, there is also evidence for anti-inflammatory and reparative properties of neutrophils in other, non-cardiovascular inflammatory conditions, including bacterial infection and non-sterile injury. Phagocytic removal of bacteria and dead cells by neutrophils clears the wound for rebuilding and repair, and depletion of neutrophils from inflamed tissue has been shown to increase the pathology, e.g., in the setting of intestinal inflammation (2, 10).

This proposed “healing” role of neutrophils is novel and somewhat unexpected in the setting of myocardial ischemia, given that neutrophils have been mostly considered exclusively pro-inflammatory by contributing to myocardial tissue damage. Nevertheless, the contribution of neutrophils in lethal reperfusion damage is controversially discussed in the literature, and attempts to develop anti-neutrophil therapies have been largely unsuccessful (11, 12). Though, it is difficult to reconcile this novel perception of neutrophil properties with clinical and epidemiological data suggesting a correlation between neutrophil blood counts and worsening of clinical outcomes in ischemic myocardial diseases. When reviewing the existing clinical evidence, however, it becomes clear that this is not a simple linear association. Only in patients with high white blood cell and neutrophil blood counts, this circulating parameter correlates with a worsened outcome (13–16).

### Neutrophil Origin, Maturation, and function

Neutrophils are recognized as first leukocytes to be recruited to an inflammatory site in order to eliminate pathogens. Their antimicrobial defense mechanisms include phagocytosis, degranulation and neutrophil extracellular trap (NET) release (17). Owing to their essential innate immune function and limited lifespan, a constant production of neutrophils from hematopoietic progenitors occurs in the bone marrow, which is named ganulopoiesis. Hematopoietic stem and progenitor cells reside in specific niches in the bone marrow, which are attracted by the stromal cell-derived chemokine CXCL12. The production of this chemokine is regulated by adrenergic nervous system signaling sensed by stromal niche cells (18). Proliferative neutrophil progenitor cells give raise to non-proliferative immature and mature neutrophils with distinct transcriptomic and functional signature (19). The key cytokine that regulates this process is granulocyte colony-stimulating factor (G-CSF). This cytokine is produced in response to interleukin (IL)-17 released by γδT cells, which in turn is controlled by IL-23 production in tissue-resident macrophages and dendritic cells (2, 20). After maturation, neutrophils are retained in the bone marrow through CXCR4 chemokine receptor signaling, and CXCR2 signaling drives their release into the circulation (21). Increased extramedullary granulopoiesis in the spleen has been shown during infection or cancer and is associated with elevated numbers of immature neutrophils in the circulation (19).

As opposed to the well-established fact that neutrophils rapidly infiltrate tissues upon infectious or sterile injury, it has been only recently appreciated that neutrophils are also found in multiple naïve tissues (22). Most tissues display circadian patterns of neutrophil infiltration, which is regulated by both tissue microenvironmental and cell-autonomous oscillations of pro-migratory factors (22, 23). Fresh neutrophils released from the bone marrow into the blood express high CD62L, which is progressively reduced on aging neutrophils, while the surface expression of CXCR4 increases prior to their clearance from the blood (24).

### Detrimental Effects of Neutrophils in Experimental Models of Myocardial Ischemia and Reperfusion Injury

Timely interventional reperfusion after the ischemic insult restores blood and oxygen supply. Yet, reperfusion exacerbates the pro-inflammatory response and further damages the initially ischemic myocardium (25). A major contributor to the ischemia/reperfusion (I/R) injury are the infiltrating neutrophils, especially at the ischemic border zone, which produce and release reactive oxygen species, resulting in acute inflammation and cardiomyocyte apoptosis (11). These processes culminate in deterioration of cardiac tissue and function. Experimental studies directly targeting abundance or function of neutrophils clearly demonstrated neutrophils as crucial contributors to I/R injury (Table 1). Approaches targeting neutrophil function mostly focus on blockade or depletion of myeloperoxidase (MPO). MPO is produced predominantly by neutrophils and Ly6C^high^ monocytes. Once infiltrated into the infarct zone, neutrophils and Ly6C^high^ monocytes release the granule heme-enzyme MPO into the extracellular space, resulting in the generation of cytotoxic aldehydes, oxidative stress, activation of enzymes degrading the extracellular matrix and further leukocyte infiltration (30). These processes eventually culminate in progressive maladaptive remodeling (27, 30). Complete MPO depletion has been reported to improve long-term outcome (24 days) post-I/R by reducing left ventricular dilatation und dysfunction with no effect on the infarct size *per se* (27). In agreement with these observations, without affecting infarction expansion, PF-1355, an MPO inhibitor, administered orally for 7 days post-I/R, mitigated acute inflammation and left ventricular dilatation (26). A longer treatment period could even restore cardiac function (26). The lack of infarct size limitation concurs with clinical trials, in which neutrophil adhesion was diminished by blockade of integrin receptors in patients subjected to angioplasty or thrombolysis (31, 32).

**Table 1:** Experimental studies targeting neutrophil counts or function in post MI healing and remodeling.

**MI model**	**Experimental** **strategy**	**Animal** **species**	**Animal** **gender**	**Experimental** **outcome**	**References**
I/R	MPO inhibition 30 min/7d, 21d I/R	C57/BL6J mice	Male	Infarct size = Leukocyte infiltration↓ Leukocyte proliferation↓ LV dilatation↓ LV function ↑	(26)
I/R	MPO deletion 30 min/24 h I/R	C57/BL6J mice	Male	Infarct size = LV dilatation↓ LV function ↑	(27)
I/R	Neutrophil depletion 45 min/3d I/R	C57/BL6J mice	Female	Infarct size↓ Ly6C^high^↓ MerTK^high^ macrophages↓	(9)
I/R	Neutrophil depletion 90 min/24 h I/R	Mongrel dogs	Male	Infarct size↓	(28)
I/R	Neutrophil depletion 4 h/6 h I/R	Mongrel dogs	Male	Infarct size =	(28)
Permanent	MPO deletion Endpoint 3, 21d post-MI	C57/BL6J mice	Male	Leukocyte infiltration ↓ LV dilatation↓ LV function ↑ PAI-1 inactivation↓	(29)
Permanent	Neutrophil depletion Endpoint 1, 3, 5, 7, 14d post-MI	C57/BL6J mice	Female	Ly6C^high^↓ M2c↑ Apoptotic cell clearance↓ LV fibrosis↑ LV function ↓	(9)

*LV, left ventricular; MPO, myeloperoxidase; PAI-1, plasminogen activator inhibitor 1*.

Other experimental anti-inflammatory strategies reporting beneficial effects on I/R injury most commonly involve interference with cardiac neutrophil recruitment. In this context, Zhang et al. recently reported reduced infarct sizes, less left ventricular fibrosis and ameliorated recovery of cardiac function following I/R in mice when deleting endothelial brahma related gene 1 (BRG1, a chromatin remodeling protein). This cardio-protection was attributed to BRG1-mediated blocking of neutrophil-endothelium adhesion and the subsequently reduced infarct-infiltration of neutrophils (33). Improved post-I/R outcome in mice deficient for plasminogen activator inhibitor-1 has also been related to reduced extravasation of neutrophils, subsequently less damaged endothelial junctions and post-ischemic micro-vascular leakage (34). Furthermore, blocking CC motif chemokine (CCL) 5-CXC motif chemokine receptor (CXCR)4 interaction in a myocardial I/R model reduced infarct size and preserved heart function, which again was associated with reduced abundance of neutrophils and prevention of neutrophil extracellular trap formation (35). These above described experimental approaches, as well as various other experimental studies comprehensively reviewed elsewhere (11, 36), suggest that interfering with cardiac neutrophil recruitment or function in experimental I/R injury might improve the cardiac outcome. However, other studies failed to show a reduction of infarct size or other beneficial effects of neutrophil-targeting approaches in cardiac I/R models. Thus, the contribution of neutrophils in lethal reperfusion damage is controversially discussed in the literature, and attempts to develop anti-neutrophil therapies have been largely unsuccessful (11, 12).

### Experimental Evidence for Protective Functions of Neutrophils in Cardiac Remodeling

In acute inflammation, neutrophils are not only vital for the clearance of pathogens or debris, but also for the resolution of inflammation and return to tissue homeostasis (2). Macrophages engulfing apoptotic neutrophils activate an anti-inflammatory response by inhibiting pro-inflammatory cytokines and inducing the production of interleukin (IL)-10, transforming growth factor-β and pro-resolving lipid mediators (12). Consequently, anti-inflammatory strategies reducing neutrophil influx in order to limit acute post-ischemic tissue injury might also inhibit the subsequent healing response. In a chronic MI model induced by permanent left anterior descending (LAD) coronary artery ligation, neutrophil-depletion resulted in a worsened cardiac function, increased fibrosis, and a progressive increase in biomarkers associated with heart failure (Table 1) (9). This was accompanied by reduced cardiac expression of myeloid-epithelial-reproductive tyrosine kinase (MerTK) on macrophages, a phagocytosis receptor that mediates the clearance of apoptotic cardiomyocytes (37). The induction of MerTK expression and increased efferocytosis capacity of macrophages was mediated via neutrophil-borne secreted factors, in particular neutrophil-gelatinase associated lipocalin, which was confirmed *in vitro* and *in vivo* (9). Beyond their established proinflammatory role in acute post-MI injury and I/R damage, this study suggests that neutrophils are pivotal modulators of the healing and remodeling after MI and consequently cardiac function. This novel role for neutrophils should be taken into account when designing and applying anti-inflammatory treatments in the setting of MI. It may explain, at least partially, why attempts to translate anti-inflammatory strategies from experimental studies into the clinical practice have been unsuccessful so far. In the future, more targeted strategies, potentially based on single molecule resolving mediators rather than broad anti-inflammatory therapies might be more beneficial.

### Circadian Rhythms Affect Neutrophil Recruitment and MI

Experimental data further suggest that the time-of-day of ischemia onset is a critical determinant when considering anti-inflammatory treatments for improving MI outcome. Mice subjected to MI at the beginning of the active phase have much larger infarcts than mice with MI induced during the resting phase (38). Epidemiological studies show a day/night pattern in the occurrence of MI and other acute cardiovascular events, with a peak in the morning and trough at night (39). Scheer et al. monitored cardiovascular parameters of 12 healthy subjects at rest and during exercise. They found that circadian rhythms in circulating cortisol, sympathetic activity, cardiac vagal modulation, arterial blood pressure and platelet reactivity mostly match the day/night patterns in adverse cardiovascular events (40). There are also clinical data supporting the notion that the time-of-day of the ischemic event onset is a critical determinant of the infarct size (evaluated by creatine kinase, CK) and mortality in ST-elevation MI patients (41–44). For example, Fournier et al. reported an independent correlation between the infarct size of ST elevation MI (STEMI) patients undergoing primary percutaneous coronary intervention (PPCI) and the time of the day at which symptoms occurred (41). A total of 353 consecutive patients was included in this study, while excluding patients with symptom-to-first-medical-contact time >12 h and patients for whom time of symptom onset was unknown. The time of symptom onset was divided into four time groups (00:00–05:59, 06:00–11:59, 12:00–17:59, and 18:00–23:59). At multivariable analysis, there was a statistically significant difference between peak CK levels among patients with symptom onset between 00:00 and 05:59 when compared with peak CK levels of patients with symptom onset in any other time group. Moreover, 30 days mortality for STEMI patients with symptom onset occurring between 00:00 and 05:59 was significantly higher than any other time group. The retrospective studies from Reiter et al. and Suarez-Barrientos et al. additionally support a circadian dependence of the infarct size and left ventricular function after STEMI on the time of symptom onset (42, 43). Circulating leukocytes oscillate between blood and peripheral tissue (45–47). In addition to leukocyte intrinsic circadian expression patterns of genes involved in cell migration, adrenergic signaling through β-adrenoreceptors on non-hematopoietic cells regulates circadian oscillations in the expression of adhesion molecules and chemokines (46, 48). The circadian fluctuations in immune cell trafficking into tissues coincide with sensitivity to acute inflammatory stimuli, being highest at the beginning of the active phase. Neutrophil production and retention in the bone marrow is time-of-day dependent (24), and circulating neutrophils at the beginning of the active phase have higher capacity to migrate into the myocardium (5). Experimental MI induced at this time point resulted in significantly higher cardiac neutrophil infiltration (5). This time-of-day-dependent extend of neutrophil recruitment into the myocardium is mediated by circadian oscillations of the chemokine receptor CXCR2 on neutrophils. Consequently, an ischemic event occurring during the active phase resulted in an exaggerated inflammation and worsened cardiac repair. Limiting neutrophil counts at this time point reduced the infarct size and improved cardiac function. This suggests that there is a threshold for neutrophil numbers recruited to the ischemic myocardium which may promote beneficial cardiac healing: although neutrophils are required for promoting monocyte recruitment, clearance of cellular debris in the infarct zone and resolution of inflammation (9), an excessive amount of neutrophils above this threshold will be detrimental (5). In conclusion, we hypothesize that there is a critical balance for neutrophils in post-MI outcome, as established previously for monocytes and macrophages by Matthias Nahrendorf et al. (4).

### Does the Ischemic Heart Recruit Distinct Neutrophil Subsets After MI?

The dual role of neutrophils in acute MI damage and healing raises the question whether the existence of distinct neutrophil subsets may provide a reasonable explanation for these controversial effects. The heterogeneity of neutrophils was originally reported in the context of infection and cancer (49, 50). In analogy to the M1 and M2 paradigm of macrophages, neutrophil subsets were named N1 and N2. N2 neutrophils were characterized as pro-tumorigenic, as opposed to a population of neutrophils within the tumor that exhibited a pro-inflammatory profile and that was named N1 (50). Ma et al. reported the existence of N1 and N2 neutrophils in the infarct region, based on gene expression profiling of isolated cardiac neutrophils using a set of pro- and anti-inflammatory markers (51). The peak of pro-inflammatory markers [such as interleukin (IL)-1β, tumor necrosis factor (TNF)α, CCL3] expressed by isolated neutrophils was at day 1, whereas anti-inflammatory markers peaked at day 5–7. The relative distribution of N1 vs. N2 neutrophils was further determined based on flow cytometric analysis of Ly6G^+^CD206^−^ vs. Ly6G^+^CD206^+^ cells. Although the majority of neutrophils detected in the infarct region 1 to 7 days post-MI was CD206 negative, a relative increase of the CD206^+^ N2 neutrophil subset up to day 7 was shown. CD206^+^ neutrophils were not found in the circulation of infarcted mice, which is in line with previous reports that the tissue environment is sufficient to promote and maintain CD206 expression on resident macrophages without the need of IL4 signaling (52). CD206, also termed as MRC1 (C-type mannose receptor 1), is mostly known as M2 marker and is a 175-kDa type I transmembrane glycoprotein which binds and internalizes glycoproteins and collagen ligands. The underlying mechanism of CD206 upregulation on this identified subset of cardiac neutrophils during the late inflammatory and resolving stage of post-MI inflammation remains unclear (51). An even more important question is whether this Ly6G^+^CD206^+^ subset exhibits distinct functional properties, in particular whether Ly6G^+^CD206^+^ neutrophils have less pro-inflammatory activity, while possibly exhibiting pro-angiogenic or pro-fibrotic actions. In support of this possibility, proangiogenic neutrophil subsets have been already described in the context of transplant neovascularization and cancer (53, 54). In an experimental mouse model of human vascular network implantation, neutrophils where identified as indispensable in proper engraftment (55). The gene expression analysis of vascular graft-derived neutrophils revealed a less pro-inflammatory, but increased anti-inflammatory and pro-remodeling expression pattern. Based on Pearson's correlation analyses comparing N1 and N2 neutrophils to various LV function variables, Ma et al. found that thinning of the LV posterior wall in systole correlated positively with N1 and negatively with N2 neutrophil polarization (51).

As to their developmental origin, it is unclear whether neutrophil subsets develop from a single precursor, and what could be the triggers for their conversion into distinct subsets. It is also conceivable that these subsets may simply reflect a more immature stage of neutrophils that are mobilized into the circulation (2, 19). Under steady-state, the majority of neutrophils is stored in the bone marrow and readily released in response to tissue injury, such as MI. Within the first few hours after MI, the bone marrow rapidly shifts from steady-state hematopoiesis to emergency granulopoiesis by enhanced proliferation of neutrophil precursors in order to respond to the increased demand of neutrophils and to replenish the bone marrow reservoir (56). Interestingly, lymphocyte activation within pericardial adipose tissue is involved in the induction of bone marrow granulopoiesis after MI, by releasing cytokines and growth factors, such as GM-CSF and IL17 into the circulation (57). Whether the pericardial lymphocyte response has an impact on the functional activity of neutrophils released from the bone marrow at different stages after MI deserves further investigation.

### Emerging Insights in Sex-Specific Differences of MI Repair and Inflammatory Responses

#### Sexual Dimorphism in MI Incidence and Outcome

While cardiovascular anatomy and physiology are quite similar in both male and female, sex exerts a considerable impact on cardiac electrophysiology and responses to pathological stimulation, such as ischemia and inflammation (58). This might not be surprising since the mammalian myocardium expresses androgen as well as estrogen receptors and is therefore sensitive and responsive to changes in the body's sex hormone household (59, 60). According to statistical evaluations, women experience their first MI at the average age of 72, which is 7 years later than men (61). Once the heart had suffered an infarction, female patients show attenuated maladaptive remodeling and ameliorated adaptation, i.e., less pronounced left ventricular wall thickening and cardiomyocyte hypertrophy (62). Additionally, insights from post-mortem and post-transplantation analyses of human myocardium revealed less cardiomyocyte apoptosis in the peri-infarct area and less necrosis in the ischemic region of female hearts (63). The reduced loss of cardiomyocytes was associated with a later transition to cardiac dysfunction and subsequent heart failure in the female donors. Several clinical as well as experimental studies have attributed the aggravated outcome after I/R to the low testosterone levels in the affected elderly men. However, testosterone replacement therapy has yielded conflicting results, with some pre-clinical and clinical studies stating beneficial effects and others reporting an inverse relationship between testosterone levels and cardio-protection (64, 65). So far, no definite benefit from the therapeutic replacement of testosterone or estrogen in primary or secondary prevention has been proven (58).

In agreement with clinical observations, animal studies show in general a better long-term outcome post-MI in female mice. In detail, females exhibit less extensive infarction expansion, left ventricular dilatation and adverse cardiomyocyte hypertrophy, paralleled by a more preserved left ventricular function (66–69). These sex-specific differences in the long-term outcome most likely result from sex-dependent distinctions in the early, acute phase of experimental MI. During that stage, males show higher incidence of cardiac rupture and therefore higher mortality rates (67–69). Furthermore, ovariectomized mice and rats have even bigger infarcts and more severe cardiac dysfunction upon I/R than male rodents. This phenotype could be partly rescued by chronic estrogen supplement (70, 71). These observations suggest a crucial role for levels of, and most likely ratios between, androgens, estrogen, and progesterone in the regulation of post-MI remodeling. Maladaptive remodeling following MI encompasses three prominent closely linked and co-dependent progressive processes: hypertrophy, inflammation and fibrosis. Human data from hypertrophied (non-ischemic) patients, experimental hypertrophy models and recent systems biology approaches revealed gender-specific alterations in LV hypertrophy, pro-inflammatory and pro-fibrotic gene expression and collagen deposition (72–74). These findings combined with conflicting observations regarding the effects of steroids on risk for or outcome post-MI imply the necessity to further elicit gender-differences in the type and extent of the different remodeling sub-processes.

#### Sex-Specific Differences in Post-ischemic Inflammation and Cellular Signaling In Neutrophils

During the 1980's and 1990's, the first discussions about a potential sexual dimorphism in inflammation emerged. Meanwhile, sex is assessed a crucial regulatory factor in various inflammatory diseases with some showing a higher prevalence in women, some in men. Yet, not only the susceptibility to a certain inflammatory disease may be sex-dependent, but also the efficacy of therapeutic strategies. Therefore, basic research outcomes as well as diagnostic procedures—based on biomarkers, disease prevention strategies, and treatment regimens in the clinic require validation in both male and female.

Sexual dimorphism in the onset, extent, progression, and resolution of inflammatory responses seem to commonly involve distinctions in neutrophil function and trafficking. Immune responses upon infection or vaccination for example are less pronounced and resolved faster in women, as determined by reduced neutrophil activation and elevation of the D-resolvin pathway compared to men (75). Additionally, female patients die less frequently from an inflammation, as severe as sepsis, than men do. This phenomenon is correlating with lower IL-6 plasma levels in women (76–78). The more favorable outcome of women after a septic shock may be predominantly associated with a gender-specific impact on neutrophil trafficking and function. In this context, it has been shown that young female peripheral blood neutrophils secret less TNFα and exhibit reduced mitogen-activated protein kinase activation *per se* and upon lipopolysaccharide stimulation than neutrophils from young men. Besides, interferon-γ triggered neutrophil priming is more pronounced in neutrophils from males than from females and accompanied by a higher expression of the pathogen-recognition receptor toll-like receptor 4 on the neutrophil surface (79). Those effects are suggested to be estradiol-dependent. A further anti-inflammatory effect of estradiol seems its induction of annexin A1 expression on neutrophils and the subsequent blockade of neutrophil-endothelial cell adhesion (77).

Cytokine production and neutrophil infiltration following endothelial adhesion represent the first inflammatory response of the myocardium after an ischemic insult. Following experimental I/R female rats exhibited lower myocardial expression and coronary effluent level of the cytokines IL-1β, IL-1α, IL-6, and TNFα and a reduced cardiac p38 activation, culminating in better restoration of cardiac function compared to males (80). MI studies in mice confirmed that males resolved IL-6 more slowly from plasma, and that male infarcted tissue showed higher IL-1β, IL-6, and IL-6 receptor expression at day 1 and 4 post-MI. Yet, no sex-differences in TNFα production were reported (69, 81). Moreover, the phenomenon of higher mortality rates in male mice post-MI was associated with a male disposition for increased neutrophil infiltration at the ischemic border zone (68, 81, 82). Infiltrating neutrophils secret matrix metalloproteinase 9 (MMP9). Increased release and activation of this protease at the site of injury culminates in infarction expansion and cardiac rupture in male rodents (68, 81, 82). Elevated MMP9 gene expression in males could also be verified in PBMCs at day 4 post-MI (the time point evaluated as the time of maximal cardiac rupture risk), but not females (81). The lower neutrophil counts in females was followed by a higher macrophage abundance at day 4 after MI (68). Hence, leukocyte ratios during post-MI inflammation and wound healing kinetics seem to be more favorable in females. These studies clearly hint toward a benefit in females encompassing the prevention of excessive post-MI inflammation and an accelerated inflammation resolution by affecting neutrophil infiltration and function. In agreement with these studies, a recent publication reported that young females had an overall lower mortality rate, less incidence of cardiac rupture, attenuated LV dilatation and an accelerated IL-6 plasma resolution than males (69). In this study, in the absence of any differences in infarct sizes at day 1 post-MI, females exhibited 2-fold lower mRNA expression of IL-1 receptor 1, IL-6 receptor α subunit, IL-13, CXCR3, CXCL4, and TIMP-1 in the infarct area. Both gender showed comparable neutrophil infiltration kinetics, yet, male myocardium showed a higher neutrophil abundance at day 3 post-MI. Besides a gender-impact on neutrophil infiltration, the researchers detected gender-differences in the phenotype of neutrophils, found in the infarcted tissue. They revealed that female neutrophils were rather polarized to the anti-inflammatory phenotype N2 with a higher capacity for the clearance of necrotic tissue, whereas, male neutrophils showed higher expression of markers characteristic for the pro-inflammatory N1 phenotype (69). Furthermore, by use of isolated human neutrophils, male neutrophils were shown to clear necrotic tissue through CD36-dependent MMP-9 degranulation while female neutrophils mainly clear cell debris via phagocytosis.

Not only sex in general, but also the menstrual cycle exerts already slight effects on the phenotype of circulating neutrophils, with diminished MMP-9 and TNFα expression during the peri-ovulatory state (83). However, most studies are performed in young (pre-menopausal) adult mice, neglecting the human situation where rather elderly patients are affected by MI and require therapies adapted to hormone household, cell renewal, proliferation and regeneration capacities altered by age. DeLeon-Pennell et al. investigated sex-differences in post-MI wound healing and remodeling in both male and female, in young and old mice, aged 3–9 months (pre-menopausal females) and 11–36 months (post-menopausal females), respectively (69). In this study, aging in females abolished the initial protection against excessive IL-6 production and signaling due to reduced liver X receptors/retinoid X receptor LXR/RXR activation. This age-dependent effect could be verified in plasma from women included in the Jackson Heart Study.

## Conclusions

There is emerging evidence for distinct neutrophil functions in the process of post-MI inflammation, resolution and remodeling, although there are still many open questions regarding their function in homeostatic and pathophysiological states that deserve further investigation. However, most of the data that we have summarized in this review are based on experimental mouse models. Dissecting the function of neutrophils in the setting of post-MI inflammation and repair will require further research in additional preclinical animal models as well as data from human samples, wherever applicable. A better knowledge of the inflammatory processes involved in MI healing may help developing more targeted therapies in order to improve the clinical outcome. In particular, stimulating the post-inflammatory resolution phase toward a well-balanced reparative response with selective mediators, potentially derived from phagocytes or the injured myocardium itself, may hold great promise. The identification of such mediators might be facilitated by the use of proteomic and lipidomic screening approaches with inflammatory cell secretome or cardiac tissue isolated after MI. Moreover, the implementation of novel techniques based on single cell RNA sequencing now allows transcriptomic analysis at a single cell level, thereby offering a valuable tool for dissecting the cellularity of the ischemic myocardium and genetic variability of individual leukocyte clusters (84, 85). Finally, it is important to consider sex-specific differences and the time-of-day of ischemia onset both in experimental as well as in human studies focusing on the link between inflammatory markers and cardiac outcome after MI.

## Author Contributions

All authors listed have made a substantial, direct and intellectual contribution to the work, and approved it for publication.

### Conflict of Interest Statement

The authors declare that the research was conducted in the absence of any commercial or financial relationships that could be construed as a potential conflict of interest.

## Abbreviations

CCL: CC motif chemokine
CXCR: CXC motif chemokine receptor
IL: interleukin
I/R: ischemia and reperfusion
LAD: left anterior descending
MerTK: myeloid-epithelial-reproductive tyrosine kinase
MI: myocardial infarction
MMP: matrix metalloproteinase
TNF: tumor necrosis factor.
